# Enabling Fresh-Produce Availability in Long-Term Care via Hydroponic Green Walls: A Multisite Operational and Cost Evaluation

**DOI:** 10.3390/nu18152553

**Published:** 2026-08-04

**Authors:** Shannon Freeman, Santiago Otalvaro Zapata, Emma Rossnagel, Atefeh Panahi

**Affiliations:** 1 Centre for Technology Adoption for Aging in the North, Prince George, BC V2N 4Z9, Canada; otalvaro@unbc.ca (S.O.Z.); rossnagel@unbc.ca (E.R.); panahi@unbc.ca (A.P.); 2 School of Nursing, University of Northern British Columbia, 3333 University Way, Prince George, BC V2N 4Z9, Canada; 3 School of Health Sciences, University of Northern British Columbia, 3333 University Way, Prince George, BC V2N 4Z9, Canada

**Keywords:** hydroponics, long-term care, nutrition, food security, nursing home, elderly

## Abstract

Background/Objectives: Limited access to fresh produce and persistent nutrition risk in long-term care (LTC) can affect dietary quality and rehabilitation capacity, particularly where seasonality and supply constraints affect food environments. Indoor hydroponic systems may support on-site production of leafy greens and herbs, but their adoption depends on practical feasibility. This study evaluated the routine staffing time and labour-only costs associated with maintaining hydroponic green wall units in LTC settings. Methods: A prospective, multi-site observational quantitative service evaluation was conducted from March 2023 to September 2024 across four LTC settings in Western Canada. Staff and volunteers logged routine interactions with wall-mounted hydroponic green wall units using adjacent mounted iPads. Entries captured task type(s), duration, using prespecified time categories, and staff role. Descriptive analyses summarized entries and actions by role and site. Labour costs were estimated using province-specific wage bands. Workload and costs were normalized using active weeks as weeks with ≥1 logged interaction. Results: A total of 112 log entries were recorded, corresponding to 362 logged actions and 962.0 min of recorded staff engagement time. Recreation therapists/dietitians submitted 45.5% of entries (*n* = 51), followed by care aides (27.7%, *n* = 31), volunteers (17.0%, *n* = 19), management (7.1%, *n* = 8), and registered nurses (2.7%, *n* = 3). Actions were dominated by monitoring and routine maintenance; 36.0% of time-recorded interactions were 0–5 min. Mean engagement was 8.7 min per time-recorded entry. Normalized to 59 active site-weeks, average workload was 16.3 min per active site-week (range 11.8–21.3), with labour-only costs of approximately 7.24 CAD per week (5.96–8.34) and 28.9 CAD per 4-week month (23.84–33.36). Conclusions: Routine upkeep of hydroponic green wall units required modest, predictable staff time and low labour-only costs, with responsibilities largely distributed across non-registered nurse roles. These findings provide feasibility parameters relevant to sustaining hydroponics as an enabling infrastructure component of nutrition-supportive LTC food environments and may be of value to inform future outcome-focused evaluations incorporating production, utilization, nutrition and rehabilitation-relevant endpoints.

## 1. Introduction

Aging brings changes in body composition, appetite, and taste that can impact nutritional needs over time [1]. Older adults depend on adequate nutrition to promote health and preserve function, support chronic disease management and treatment, and participate effectively in rehabilitation [2]. In institutional contexts such as long-term care (LTC) facilities, this imperative translates into building food environments that reliably deliver palatable, nutrient-dense options that meet dietary and nutritional needs [3]. International consensus guidelines and evidence syntheses consistently report elevated nutrition risk among residents of care facilities and recommend coordinated, food-first strategies delivered by multidisciplinary teams [4].

In Canada, estimates from LTC indicate high prevalence of malnutrition risk [5], highlighting the need for practical, facility-level strategies that improve access to appealing, nutrient-dense foods and support mealtime engagement [6]. Beyond malnutrition prevalence estimates, Canadian intake data suggest that the LTC food environment does not consistently meet residents’ micronutrient requirements, indicating a day-to-day nutritional gap that can undermine strength, mobility, and rehabilitation capacity [7]. In northern and rural regions, short growing seasons, harsh winter conditions, and logistics-sensitive supply chains constrain dependable access to fresh produce and drive higher prices—factors that erode dietary quality and variety for residents and strain food-service budgets [8].

Indoor hydroponic systems offer a facility-embedded, year-round means to produce leafy greens and culinary herbs on-site [9]. These compact, soil-free systems use recirculating nutrient reservoirs and timer-controlled LED lighting to maintain stable growth cycles independent of weather; water and nutrient use are efficient, tasks are schedulable (water-level checks, top-ups/nutrient dosing, pruning), and the absence of soil reduces some infection-control concerns in shared spaces [10]. There is substantial evidence that hydroponic and horticulture-based programs confer psychosocial benefits (e.g., reduced depressive symptoms, greater social engagement) [11], while simultaneously enabling on-site production of fresh, appealing greens and herbs that can be incorporated into menus, snacks, and therapeutic diets [12,13].

Despite growing interest in on-site food production through hydroponic systems, their integration into LTC settings depends on practical feasibility. In these resource-constrained environments, decision-makers require clear evidence of the operational demands and staffing implications of maintaining such systems [14]. This feasibility is an upstream determinant of whether hydroponic production can be maintained as a reliable input to food service, which may subsequently support nutrition-focused care for rehabilitation-eligible residents. Yet, little is known about the routine workload or staff time required to sustain hydroponic gardening in real-world care facilities [11], especially in northern and rural regions, where staffing shortages, limited resources, and implementation challenges create additional barriers to adoption [15]. To address this gap, this study evaluated the feasibility and human resource demands associated with integrating hydroponic gardening into LTC settings.

## 2. Materials and Methods

### 2.1. Study Design and Setting

**Figure 1:**
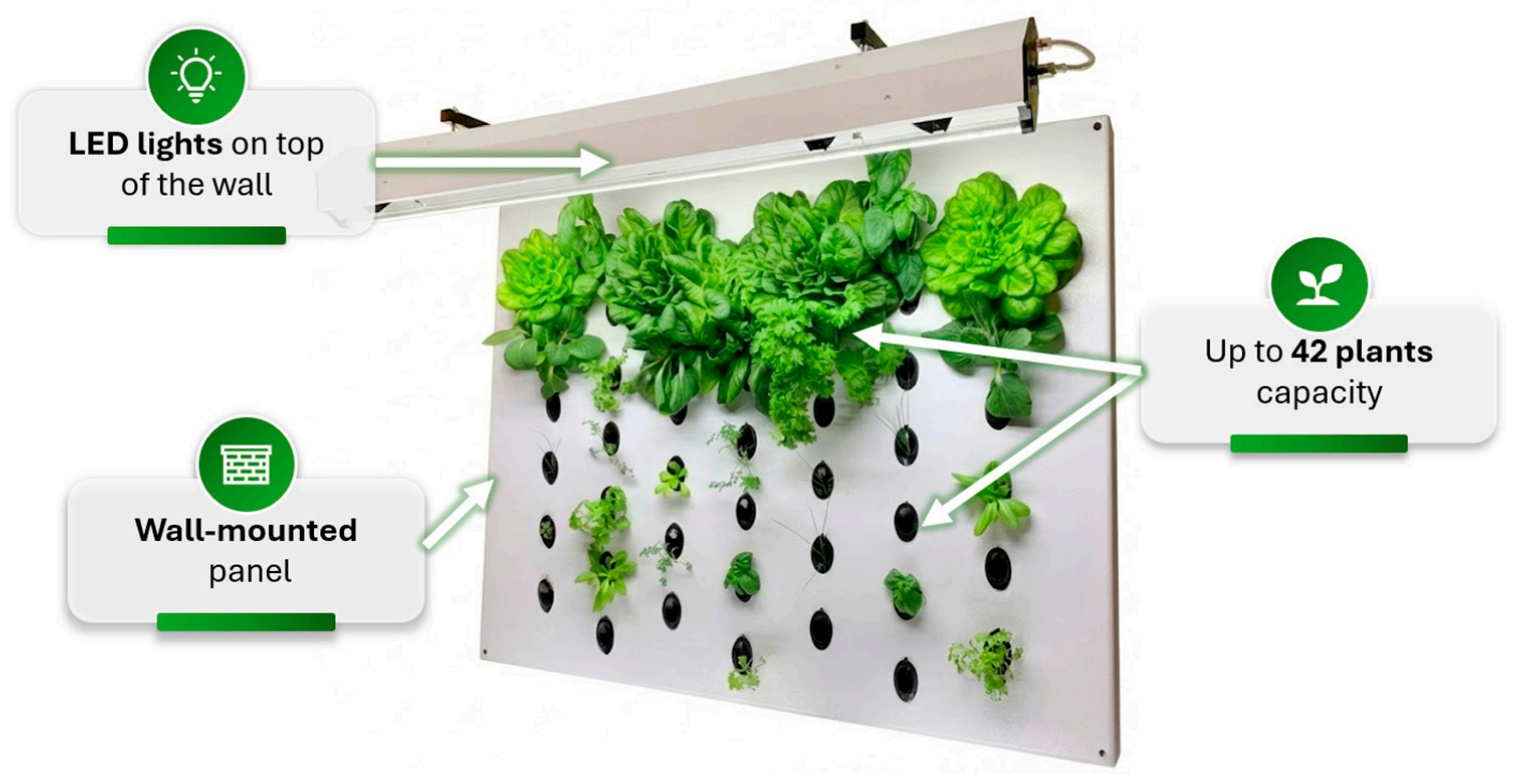
Indoor hydroponic configuration deployed across all sites: wall-mounted JustVertical Green Wall unit with overhead LED lighting and up to 42 plant sites.

A prospective, multi-site observational quantitative service evaluation was conducted from March 2023 to September 2024 across four LTC settings in Western Canada. Participating sites were identified because they had already installed, or were in the process of installing, the hydroponic gardening technology and had expressed interest in participating in the evaluation. Sites were assigned anonymized identifiers (Site 1–Site 4) for reporting purposes. Participating sites varied in facility size and care context, including two large LTC homes (>100 publicly funded beds), one smaller LTC home (~50 publicly funded beds), and one small dementia-focused supportive living setting (<20 self-contained publicly funded beds). All sites installed the same wall-mounted indoor hydroponic green wall system from JustVertical (https://justvertical.com/ see Figure 1). Placement of the hydroponic unit within each facility was determined by local leadership in consultation with the research team to support safe installation and maximize accessibility and visibility for residents, staff, and visitors. Local maintenance staff were engaged from project outset and were responsible for installation at each site.

### 2.2. Participants and Data Collection

Any staff member or volunteer who interacted with a hydroponic unit during routine activities was eligible to record an interaction log. Site leads received hands-on training and used a train-the-trainer approach to orient staff and volunteers to routine activities which included water-level checks, water and nutrient top-ups, pruning, planting, and harvesting, and were provided instruction on the interaction logging procedure. Training regarding routine activities was developed independently by each site based on the information provided with the hydroponic green wall. Instruction on the interaction logging procedures was provided in person by the research team. Sites were able to select plants according to their preferences, resident interests, and facility routines. Some plants grown across sites included herbs, lettuce, vegetables, and edible flowers. Because the systems were installed indoors, they were available for use throughout the year rather than being limited to the outdoor growing season.

Data were collected using an iPad mounted adjacent to each unit, enabling point-of-care logging at the time of interaction. The interaction log was designed as a brief, low-burden self-recording tool to support real-time completion in routine LTC workflows. To support consistent data capture across sites, the iPads were positioned directly beside the gardens at eye level, and the research team remotely monitored the devices to confirm that they remained plugged in and functioning. Site trainers were engaged throughout implementation, and any concerns regarding log completion were addressed during routine check-ins. Before submitting a log entry, users were presented with the study information letter and consent form on the iPad; only consenting individuals could proceed to complete and submit the log. For each entry, participants selected one or more task types from a predefined list, recorded the interaction duration using prespecified time categories, and identified their role within the facility. This project was reviewed and approved by the UNBC research ethics board (File No. 6009005, approved on 22 November 2022).

### 2.3. Data Analysis

Quantitative data collected on the iPads were exported to Microsoft Excel and summarized using SPSS version 30.0.0. Analyses included descriptive statistics and graphics. Crosstabs were created to compare the variables and identify the reported behaviour of the staff with the hydroponic unit. A cost analysis described the human and financial resources utilized to implement hydroponic gardening. Costs were estimated from role-specific hourly wages from the Government of Canada Job Bank in BC during 2025 [16] using the published lower, median, and upper wage bands; volunteer time was recorded but not monetized. Minutes were aggregated by role across all sites and by site across all roles. For time/cost analyses, the combined ‘recreation therapists/dietitians’ group was disaggregated into dietitians and recreation therapists due to different wage bands.

For role-level averages, the denominator was the total number of active weeks for that role across all sites (weeks in which that role recorded ≥1 interaction). For site-level averages, the denominator was the total number of active weeks for that site (weeks in which the site recorded ≥1 interaction). Base-case estimates used median wages, and lower/upper wage-band scenarios were reported secondarily. Weekly estimates were expressed as monthly values using a four-week conversion. Finally, a single-role scenario estimated monthly cost, assuming one role performed all tasks for an average of 13 min per week over four weeks. Capital purchases, consumables, utilities, benefits/overheads, and overtime were not included.

## 3. Results

### 3.1. Staff Roles and Interaction Frequencies

Across the four participating sites, a total of 112 log entries were submitted by staff and volunteers interacting with the hydroponic green wall units between March 2023 and September 2024. Roles were grouped into five categories for analysis: (1) care aides and dementia care staff, (2) recreation therapists and dietitians, (3) program coordinators and operations managers (management), (4) volunteers, and (5) registered nurses. The largest proportion of log entries came from recreation therapists and dietitians (45.5%, *n* = 51), followed by care aides (27.7%, *n* = 31), volunteers (17.0%, *n* = 19), management (7.1%, *n* = 8), and registered nurses (2.7%, *n* = 3).

**Figure 2:**
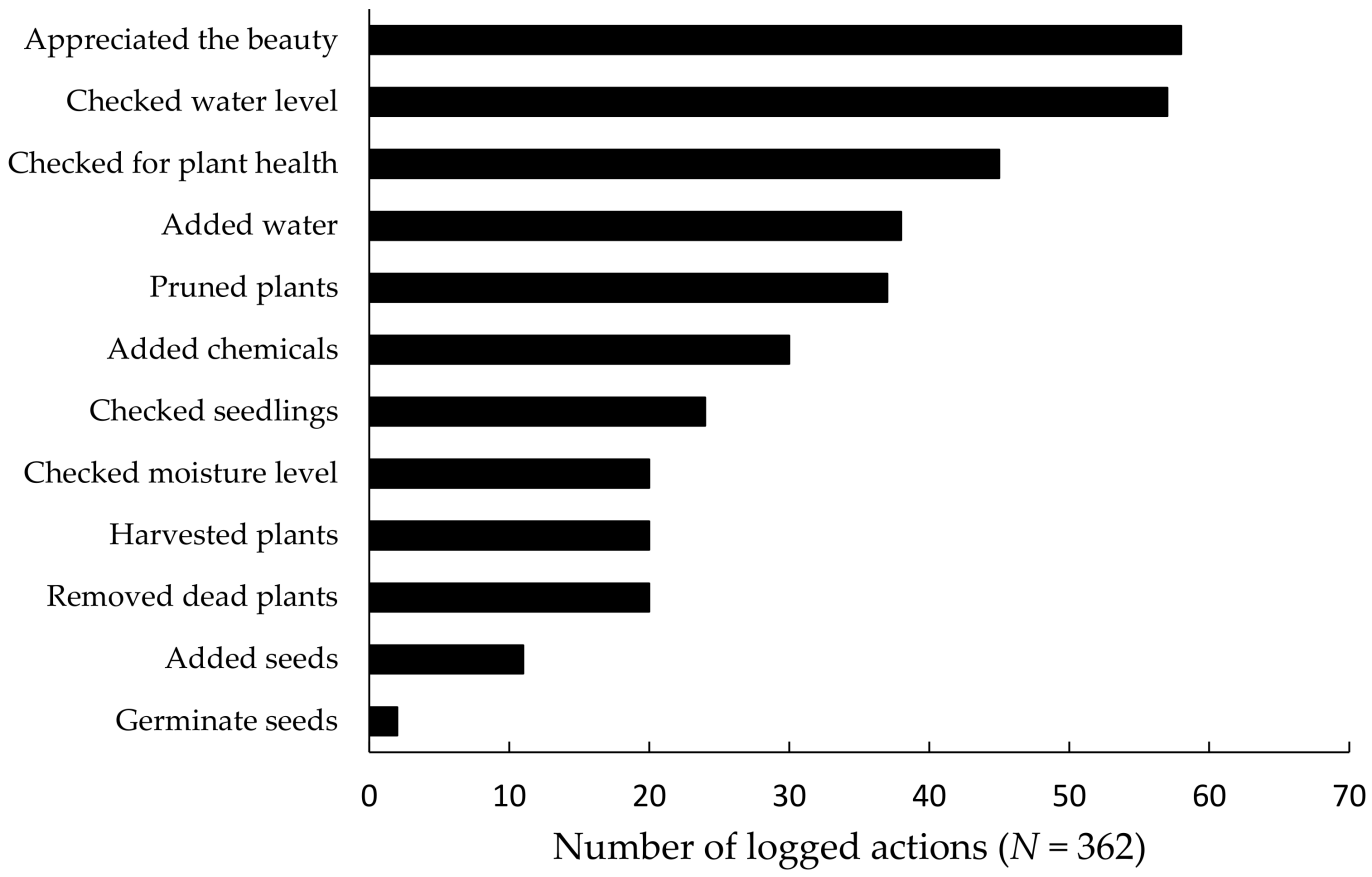
Frequency of logged hydroponic unit actions across four long-term care sites (March 2023–September 2024). Bars indicate the number of logged actions (total actions *N* = 362). Because a single log entry could include multiple tasks, action counts exceed the number of log entries (*n* = 112).

As a single log entry could include multiple tasks, 112 entries corresponded to 362 logged actions during the study period. Overall task frequencies are shown in Figure 2. The five most frequently logged actions accounted for approximately two-thirds (~65%) of all recorded actions, indicating that logged activity was concentrated in a small subset of routine tasks. Aesthetic engagement and routine monitoring (e.g., water-level checks) were most frequently logged, whereas planting-related actions (e.g., seed addition and germination) were uncommon.

### 3.2. Functional Roles and Task Categories

**Figure 3:**
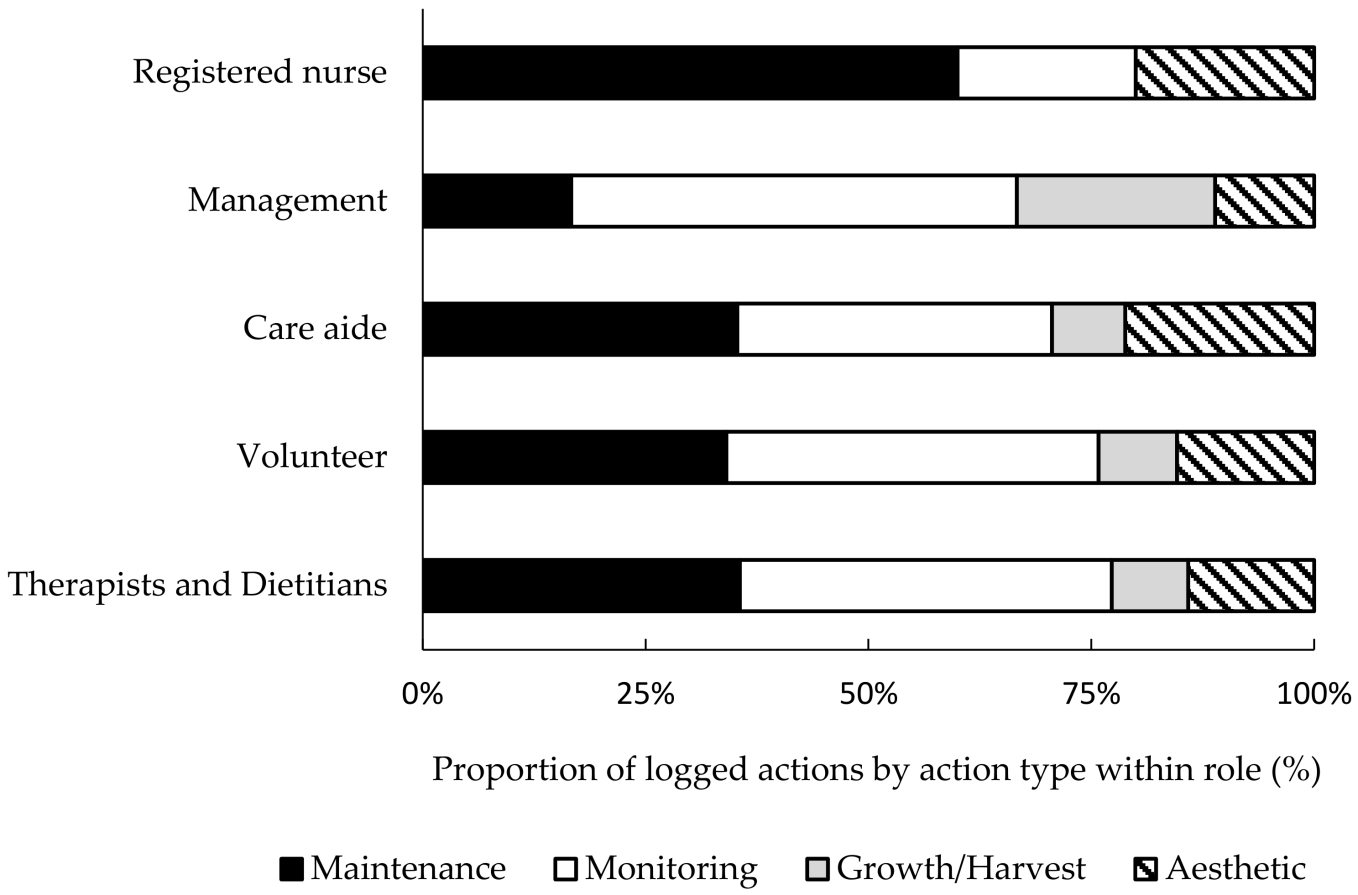
Distribution of logged actions by functional task category within staff roles. Bars represent within-role percentages across four long-term care sites (March 2023–September 2024). Total actions by role were recreation therapists/dietitians (*n* = 163), volunteers (*n* = 91), care aides (*n* = 85), management (*n* = 18), and registered nurses (*n* = 5). Interpretations for roles with low action counts should be made cautiously.

Role-specific distributions across functional task categories are summarized in Figure 3 (within-role percentages). In total, recreation therapists and dietitians recorded the most actions (*n* = 163), followed by volunteers (*n* = 91), care aides (*n* = 85), management (*n* = 18), and registered nurses (*n* = 5). Across roles, logged actions were dominated by maintenance and monitoring activities. Management entries were primarily monitoring-oriented, whereas recreation therapists/dietitians, volunteers, and care aides demonstrated a balanced mix of monitoring and maintenance. Growth/harvest activities accounted for a smaller share across roles, and aesthetic engagement was most prominent among care aides.

### 3.3. Staff Time and Cost Analysis

Time spent interacting with the hydroponic units was assessed using the time bands recorded in the log. Overall, 36.0% of time-recorded interactions were brief (0–5 min). Converting time bands to minutes using midpoint estimates, total recorded engagement time across all roles was 962.0 min, corresponding to a mean of 8.7 min per time-recorded log entry over the study period. See Table 1 for role-level engagement time and the corresponding labour cost estimates (low/median/high wage bands).

When normalized to 59 active site-weeks (weeks in which a facility recorded ≥1 interaction), this equated to an average workload of 16.3 min per active site-week across all roles. Using role-specific active weeks as denominators, recreation therapists recorded the greatest engagement time (87.4 min/month), followed by volunteers (68.7 min/month; not monetized), dietitians (60.2 min/month), care aides (47.4 min/month), operations managers (21.5 min/month), and registered nurses (10.0 min/month).

Across sites, total engagement time ranged from 59.0 to 469.5 min over the study period; when normalized by active weeks, site-level engagement ranged from 11.8 to 21.3 min per active week, corresponding to estimated costs of $5.96–$8.34 CAD per week ($23.84–$33.36 CAD per month), depending on staff mix and wage bands. Site 1 recorded the highest engagement (469.5 min across 22 active weeks; 21.3 min per active week) and the highest estimated cost ($8.34 CAD per week; $33.36 CAD per month), whereas Site 2 recorded 231.5 min across 19 active weeks (12.2 min per active week) with a lower estimated cost ($5.96 CAD per week; $23.84 CAD per month). Sites 3 and 4 showed comparable monthly costs ($30.38 and $25.13, CAD respectively) but differed in engagement intensity (15.5 min per active week vs. 11.8 min per active week). See Table 2 for a detailed description of all results across sites.

## 4. Discussion

This multi-site service evaluation quantified the routine operational workload associated with maintaining indoor hydroponic green wall units in four northern British Columbia LTC facilities. When normalized to active site-weeks, logged engagement averaged 16.3 min per active site-week across roles, with low estimated labour costs (approximately $7.24 per week; $28.94 per 4-week month). Recorded activity was largely concentrated on monitoring and routine maintenance, while planting and harvest actions were comparatively less frequent. In resource-constrained LTC environments where new initiatives must fit within competing clinical and operational priorities [17], these findings indicate that ongoing upkeep could remain modest and predictable.

Engagement with the JustVertical hydroponic gardening units was concentrated among recreation therapists/dietitians, care aides, volunteers, and management, with few recorded actions by RNs. This pattern suggests hydroponic upkeep can be embedded within existing recreation, dietary, and support-staff workflows rather than relying heavily on RN time or dedicated hydroponic green unit staffing. Similar role patterns have been described in horticulture- and garden-based initiatives in institutional care, which are commonly delivered through recreation/activity programming, dietary services, support staff, and volunteers; however, routine staffing inputs and the role distribution required for sustainment are rarely quantified in the literature [11,18]. In northern and rural LTC settings where staffing pressures and competing priorities are common [19], role alignment that distributes responsibility across multiple non-RN roles may improve implementation fit by reducing dependence on any single professional group and by supporting flexible integration into day-to-day routines [20].

More broadly, these findings speak to an implementation gap highlighted in rehabilitation nutrition for older adults: while nutrition risk is shaped by sensory, cognitive, psychological, and social vulnerabilities that often require coordinated multidisciplinary responses [21], practical evidence on how environmental strategies can be sustained within routine institutional workflows remains limited [22,23]. In this context, hydroponic green units are best conceptualized as enabling infrastructure that can support nutrition-focused processes rather than as a standalone intervention [12,24]. A key implication for geriatric rehabilitation nutrition is that low-burden on-site growing systems are unlikely to influence intake or function unless they are embedded within explicit workflows that connect production to consumption [12,25]. Examples include a standardized harvest-to-kitchen process, defined food-handling and storage procedures, and planned menu integration (e.g., using herbs and leafy greens for flavour enhancement, garnish, and snack or sandwich additions) [26,27,28]. Framing hydroponics as part of a broader “food-first” delivery model may align with the multidisciplinary emphasis in rehabilitation nutrition, where sustainable delivery systems and role clarity are prerequisites for consistent nutritional impact [4].

The task profile observed in this evaluation provides additional implementation insight. The predominance of monitoring and routine maintenance suggests that ongoing upkeep is composed mainly of brief, schedulable checks and small corrective actions, supporting the feasibility of workflow integration [25]. At the same time, the relatively low frequency of planting and harvest activity may reflect variability in how sites operationalized production, such as differences in planting responsibility, harvest practices, or the extent to which plants were grown for consumption versus primarily for engagement and display [11,12,13]. The observed differences in engagement intensity across sites are therefore best interpreted as implementation variability shaped by local conditions (e.g., staff champions, unit visibility and placement, proximity to water access, clarity of responsibility assignment, and training reinforcement), rather than as performance differences between facilities [29].

From a decision-maker perspective, the labour estimates presented here are useful as labour-only indicators of incremental workload. The direct staffing input required to sustain the units was small in absolute terms, which is meaningful in LTC settings where even minor additions to workload can be difficult to absorb [19,30]. However, the overall resource case for hydroponic integration also depends on factors not captured in this evaluation, including capital costs, consumables, utilities, infection-control procedures, and the extent to which harvested items are reliably incorporated into menus [31,32]. For that reason, future work would benefit from a cost-consequence approach that combines labour inputs with simple production and utilization metrics (e.g., harvest weight or usable yield, frequency of menu incorporation, plate waste) to clarify whether operationally feasible hydroponics can contribute meaningfully as an infrastructure component to enable nutrition-supportive care among rehabilitation-eligible residents.

### 4.1. Limitations

This evaluation should be interpreted considering several limitations. First, activity logs are a pragmatic proxy for engagement and are susceptible to under-reporting and variability in logging behaviour across roles and sites; some interactions likely occurred without being recorded. Second, interaction duration was captured using time bands and converted using midpoint estimates, introducing measurement imprecision. Third, cost estimates reflect labour only and exclude capital costs, consumables, utilities, benefits/overhead, and potential overtime; volunteer time was recorded but not monetized, which may understate total human input while reflecting direct financial costs. Finally, the study involved four LTC sites within one northern region and did not measure produce yield, menu integration, dietary intake, nutrition risk, or functional outcomes, limiting generalizability and precluding inference about nutritional or rehabilitation effects.

### 4.2. Implications and Next Steps

Despite these limitations, this service evaluation establishes feasibility parameters for hydroponic integration in LTC and highlights modifiable implementation levers that can inform rehabilitation- and nutrition-relevant research. It is noted that this study focused on evaluation of implementation feasibility and as such, a logical next step for future research is to examine deeper the clinical and nutritional effectiveness of the hydroponics. This should include pairing hydroponics with explicit, routine nutrition workflows (e.g., standardized harvest-to-kitchen processes, safe food-handling procedures, structured menu incorporation of harvested items, and snack or flavour-enhancement protocols) and to evaluate outcomes aligned with older adults who are eligible for rehabilitation. Proximal nutrition indicators could include harvest yield and utilization, menu incorporation frequency, plate waste, and nutrition risk screening, alongside rehabilitation-relevant outcomes such as participation in therapy, activities of daily living performance, and mobility indicators [21,33]. Future work should also incorporate implementation outcomes (adoption, fidelity, sustainment) and document local determinants (champions, placement, staffing models, training reinforcement) to clarify the conditions under which hydroponics can be most effectively integrated into comprehensive nutrition-supportive care in institutional settings.

## 5. Conclusions

This multi-site service evaluation provides pragmatic implementation evidence on the routine staffing footprint associated with maintaining indoor hydroponic green wall systems in LTC settings. Across diverse care contexts, upkeep was characterized by brief, recurring monitoring and maintenance tasks and was distributed primarily across recreation/dietary staff, care aides, volunteers, and management, with minimal reliance on registered nursing time. In resource-constrained settings, the observed labour requirements suggest that hydroponic systems may be operationally compatible with routine workflows, strengthening their plausibility as a feasible infrastructure component to enable nutrition-supportive food environments. Importantly, these findings reposition hydroponics from a novelty activity to a form of enabling infrastructure: the degree to which on-site growing can contribute to rehabilitation-relevant nutrition ultimately depends on whether feasible maintenance translates into reliable production and consistent integration into food service and mealtime processes. Future evaluations should therefore extend beyond labour inputs to assess production, utilization, and integration pathways (e.g., yield, harvest-to-kitchen processes, menu incorporation, and plate waste), alongside nutrition risk and functional indicators, within cost-consequence frameworks that incorporate capital and consumable inputs.

## Figures and Tables

**Table 1:** Role-level engagement time and labour cost estimates for hydroponic green wall upkeep.

Role	Minutes per Month	Low Hourly Wage Cost per Month	Median Hourly Wage per Month	High Wage Hourly Cost per Month
Recreation Therapist	87.5 min	$32.08 CAD	$46.67 CAD	$72.53 CAD
Dietitian	68.7 min	$22.80 CAD	$41.67 CAD	$57.25 CAD
Care aide	47.4 min	$15.01 CAD	$18.96 CAD	$22.78 CAD
Operations Manager	21.5 min	$10.71 CAD	$17.92 CAD	$30.52 CAD
Registered Nurse	10.0 min	$5.00 CAD	$7.21 CAD	$9.06 CAD
Volunteers	68.7 min	$0.00 CAD	$0.00 CAD	$0.00 CAD

Minutes/month were calculated using role-specific active weeks (weeks in which that role recorded ≥1 interaction) and a 4-week month conversion. Labour costs were calculated as (minutes per month ÷ 60) × hourly wage, using BC Job Bank low/median/high wage bands (Canadian dollars [CAD]/hour): Registered nurse (30/43.27/54.37), Recreation therapist (22/32/49.73), Dietitian (22.53/41.63/50), Care aide (19/24/28.84), Operations manager (29.88/50/85.16). Costs reflect labour only and exclude benefits/overhead, capital, consumables, utilities, and overtime. Volunteer time was recorded but not monetized.

**Table 2:** Staff time and labour cost for hydroponic green wall maintenance by site (per active week).

Sites	Active Weeks ^1^	Total Minutes Reported	Average Minutes per Week	Average Cost per Week	Average Cost per Month
Site 1	22 weeks	469.5 min	21.3 min	$8.34 CAD	$33.36 CAD
Site 2	19 weeks	231.5 min	12.2 min	$5.96 CAD	$23.84 CAD
Site 3	13 weeks	202 min	15.5 min	$7.60 CAD	$30.38 CAD
Site 4	5 weeks	59 min	11.8 min	$6.28 CAD	$25.13 CAD
Total	59 weeks	962 min	16.3 min	$7.24 CAD	$28.94 CAD

^1^ Active weeks are weeks with ≥1 logged interaction per site; the Total row uses active site-weeks (sum across sites). CAD denotes Canadian dollars/hour): Monthly costs are assumed for 4 weeks/month. Volunteer time was not monetized.

## Data Availability

Data are available upon request from the corresponding author.

## Abbreviations

The following abbreviations are used in this manuscript:

LTC	Long-term Care
